# Compartment-Specific Differentiation of Bacterial Communities in the Sea Cucumber *Holothuria leucospilota* from the South China Sea

**DOI:** 10.3390/microorganisms14091900

**Published:** 2026-08-27

**Authors:** Xiaoyu Chen, Xinye Chen, Jinrong Shou, Jiaojiao Zhang, Jiening Zou, Xinyi Bao, Zonghe Yu, Xiaoyong Zhang

**Affiliations:** 1 University Joint Laboratory of Guangdong Province, Hong Kong and Macao Region on Marine Bioresource Conservation and Exploitation, College of Marine Sciences, South China Agricultural University, Guangzhou 510642, China; 13640009806@163.com (X.C.);; 2 Biological Physics Laboratory, School of Physics and Astronomy, University of Manchester, Schuster Building, Manchester M13 9PL, UK; 3 College of Food and Health, Zhejiang A&F University, Hangzhou 311300, China

**Keywords:** sea cucumber, 16S rRNA gene, gut bacteria, *Holothuria leucospilota*, environmental filtering

## Abstract

While the gut bacterial communities of sea cucumbers have been extensively studied, the extent to which environmental filtering may shape bacterial assembly across different host compartments remains poorly understood. This study employed Illumina MiSeq high-throughput sequencing technology to investigate the bacterial communities in environmental sediments (E), body epidermis (B), foregut (F) and hindgut (H) samples of *Holothuria leucospilota* (n = 4 per group) from the South China Sea. A total of 749,222 high-quality 16S rRNA sequences were dereplicated into 911 amplicon sequence variants (ASVs). Alpha diversity revealed that environmental sediments exhibited the highest diversity among the four samples (Shannon index: 7.33 ± 0.11). Beta-diversity analysis revealed pronounced differentiation in bacterial community structure among sample types. Proteobacteria, Bacteroidetes, and Firmicutes were the dominant bacterial phyla. The bacterial taxonomic composition across compartments of *H. leucospilota* varied considerably. *Woeseia* was not detected from host niches but was present only in environmental sediments. The genus *Vibrio* dominated in environmental sediments and foregut samples. The epidermal bacterial communities were characterized by the prevalence of the NS11-12 marine group, Rhodobacteraceae, and Rhizobiaceae. The distinct successional gradient observed in *Woeseia*, *Vibrio*, and *Pseudomonas* suggested that bacterial colonization of the gut is consistent with selective environmental filtering. Functional annotation (KEGG) indicated dominance in core metabolic pathways, particularly carbohydrate and amino acid degradation, revealing complementary metabolic functions among dominant taxa with the potential for metabolic niche partitioning to support host energy harvest. Our findings provide a baseline framework that may assist in future screening of probiotic candidates and disease management strategies, pending experimental validation of the functional roles of the identified taxa.

## 1. Introduction

Sea cucumbers possess a simple tubular digestive tract capable of holding a large amount of sediment [1], making them quintessential marine benthic organisms. They obtain energy by consuming organic matter in the sediment, such as bacteria, diatoms, macroalgae fragments, and protozoa [2], thereby transferring nutrients from detritus to higher trophic levels [3]. However, in oligotrophic marine environments, where surface sediments are often depleted of diatoms and macroalgal detritus, sediment bacteria serve as a critical nutritional subsidy for sea cucumbers. These microbes not only provide a direct carbon source but also supply essential nutrients that are otherwise inaccessible to the host [4]. Through strong bioturbation, benthic detritivores promote undersea ecological cycles, enhance biodiversity, and perform significant ecological functions [3,5]. Biological disturbance drives increases in benthic biodiversity and biomass, which in turn act as key drivers of enhanced primary productivity [6,7]. Furthermore, sea cucumbers purify the seabed substrate, thereby maintaining the balance of the marine ecosystem and reducing the occurrence of coral diseases [8]. Given their pivotal role in benthic nutrient cycling, understanding the bacterial symbioses underlying sea cucumber health is of paramount importance.

The health and growth of the host are closely related to symbiotic bacterial communities, which may harbor multiple species of symbiotic bacteria [9,10]. Symbiotic bacteria contribute to host fitness through the biosynthesis of essential vitamins and amino acids, as well as the fermentation of complex polysaccharides into short-chain fatty acids [11]. The symbiosis between sea cucumbers and their gut bacterial communities is well-established ecological interaction [3]. Previous studies have demonstrated that Bacteroidetes excel at acquiring and digesting various nutrients such as polysaccharides, lipids, and proteins [12]. Their metabolic activity may directly or indirectly enhance carbohydrate production, thereby benefiting the host during intestinal regeneration [13]. Collectively, these findings suggest that the gut microbiome possessed regulatory mechanisms to adapt to gut regeneration after injury of sea cucumbers. Pan et al. [14] reported that cellulolytic enzymes absent from the sea cucumber genome are encoded by gut-associated bacterial communities, implying that microbial symbionts complement host-derived digestive capacity by supplying key polysaccharide-degrading activities. This complementarity between host and bacterial digestive capabilities highlights the reliance of sea cucumbers on their microbial symbionts. Given the recalcitrance of most marine microorganisms to axenic culture, high-throughput sequencing has become the prevailing approach for characterizing the taxonomic composition and functional potential of sea cucumber bacterial communities. For example, through high-throughput 16S rRNA gene sequencing and metagenomic sequencing, researchers can reveal the diversity of microorganisms in the deep-sea environment and their metabolic potential in the sea cucumber intestine [15]. However, existing studies predominantly focus on single niches (e.g., the gut) [16], largely overlooking the spatial continuum from the ambient sediment to the epidermis and through the gastrointestinal tract. Environmental filtering is a key process in the assembly of host-associated microbiomes. Yet, it remains unclear how selective barriers across different body compartments shape the taxonomic and functional profiles of *H. leucospilota* bacterial communities. This study therefore aimed to elucidate the spatial heterogeneity of bacterial communities across these compartments and to test the hypothesis that compartment-specific environmental filtering may shape bacterial assembly in this sea cucumber species. To this end, we selected *H. leucospilota*, a widely distributed and commercially valuable sea cucumber, and collected specimens from the coastal waters of Sanya (South China Sea) for this case study.

Among the diverse sea cucumbers, *H. leucospilota* represents a particularly informative system for studying marine symbiosis. *H. leucospilota* is widely distributed across the South China Sea and is commercially exploited as a high-value edible sea cucumber [1,17]. As benthic detritivores inhabiting natural marine sediments, *H. leucospilota* feeds on unprocessed organic matter without anthropogenic antibiotic exposure. Consequently, the gut bacterial communities of wild-caught specimens are likely to reflect the native microbial assemblage of natural sea cucumber populations. Elucidating the bacterial communities of *H. leucospilota* can therefore inform sustainable aquaculture and biosecurity strategies. Given the structural complexity of marine invertebrates, microbial colonization likely involves multiple tiers of selective barriers, from physical defenses at the epidermis to physiological constraints in the gut. In this study, we profile the bacterial communities inhabiting the epidermis, foregut, and hindgut of *H. leucospilota*, as well as the surrounding surface sediments, using 16S rRNA gene amplicon sequencing. This multi-compartment approach enabled us to characterize taxonomic diversity, predict metabolic potential, and determine the linkages of bacterial communities among epidermis, gut and the environmental sediments, with the goal of identifying dominant bacterial taxa and their potential links to host physiology and nutritional metabolism.

## 2. Material and Methods

### 2.1. Sample Collection and Analysis

**Figure 1:**
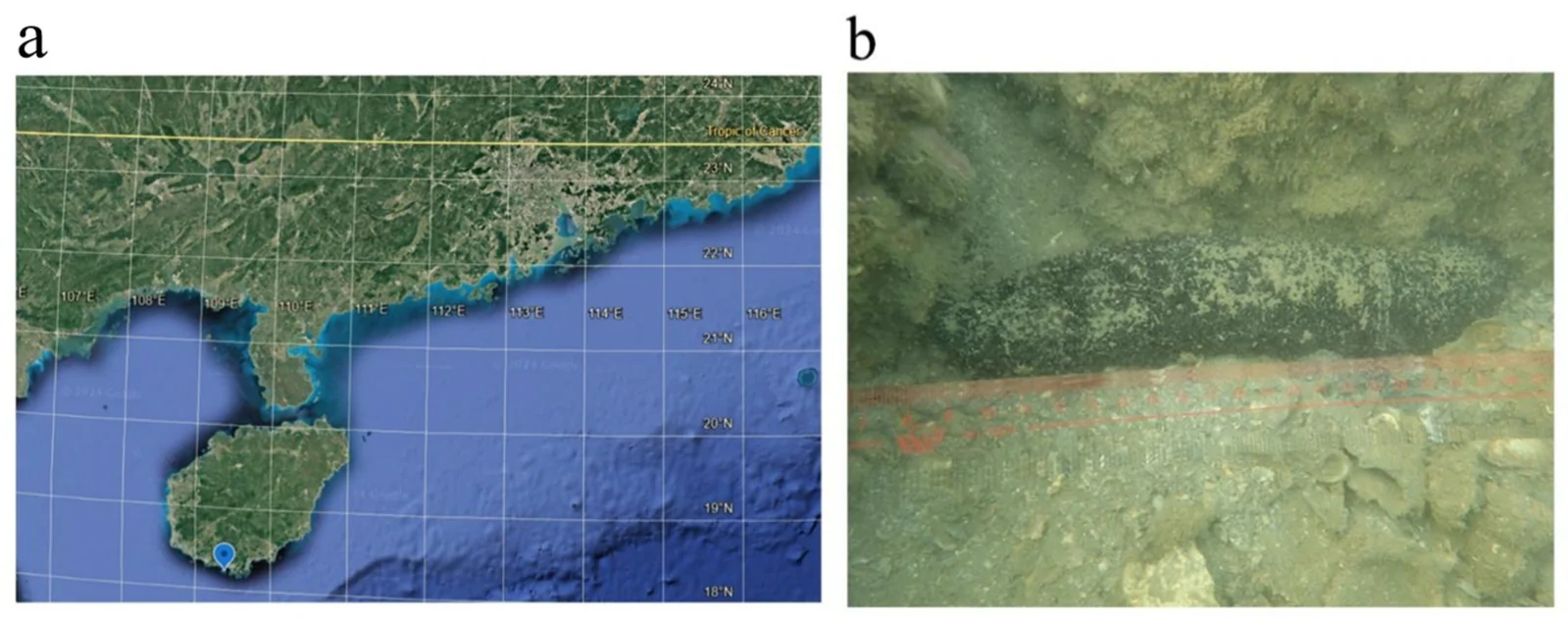
Sampling site location (**a**) and photograph of *H. leucospilota* (**b**).

In August 2024, samples were collected by diving in the coastal waters of Sanya (18° 11′ N, 109° 25′ E) (Figure 1). A total of 12 healthy *H. leucospilota* individuals of similar size and with intact epidermis were collected. Four individuals were designated for epidermis sampling, four for foregut content sampling, and four for hindgut content sampling. Surface sediment samples (0–2 cm) were collected using 50 mL sterile syringes from four sites located approximately 20 m apart, each adjacent to one of the individuals used for epidermis sampling (E1–E4) [18]. The collected specimens were immediately stored on ice and transported to the laboratory within an hour. Species identification of *H. leucospilota* was performed based on morphological characteristics following the taxonomic keys of Purcell et al. [1] and by comparing external features (body color, papillae arrangement, and calcareous ring structure) with reference specimens.

Before dissection, sea cucumber specimens were gently rinsed 2–4 times with sterile seawater to remove surface debris and silt, thereby minimizing contamination. For epidermis sampling, the body surface was carefully scraped with a sterile scalpel to obtain mucus and superficial epidermal cells (B1–B4) [19]. For gut sampling, the ventral abdomen was opened with a sterile scalpel, and the contents of the foregut and hindgut were carefully expressed separately, avoiding intestinal tissue, and collected in sterile cryotubes (F1–F4 and H1–H4) [20]. Sediment samples were designated E1–E4, corresponding to the respective epidermis individuals. In total, 16 samples were obtained from four compartments (environmental sediments, body epidermis, foregut, and hindgut), each with four biological replicates. All samples were kept at −80 °C until DNA extraction.

### 2.2. DNA Extraction and High-Throughput Sequencing

Samples were placed in sterile mortars with liquid nitrogen and homogenized thoroughly using a pre-chilled pestle. Approximately 0.5 mL of the resulting homogenate was transferred to a 0.8 mL TE buffer tube, its volume was measured by the water displacement method, and the sample was immediately processed for DNA extraction. Genomic DNA was extracted from the samples using a DNA extraction kit and its concentration was measured using the Qubit^®^ dsDNA HS Assay Kit and nucleic acid quantitative instrument (Thermo Fisher Scientific (Invitrogen), Waltham, MA, USA). Total genomic DNA from all samples was extracted using an E.Z.N.A. Soil DNA Kit (Omega Biotek, Winooski, VT, USA) following the manufacturer’s protocols [21]. The V3–V4 region of the 16S rRNA gene was amplified using forward primers containing the sequence “CCTACGGRRBGCASCAGKVRVGAAT” and reverse primers containing the sequence “GGACTACNVGGGTWTCTAATCC”. PCR amplification was performed in a 25 μL reaction with 2.5 μL of TransStart Buffer, 2 μL of dNTPs, 2 μL of forward and reverse primers, 0.5 μL of TransStart Taq DNA Polymerase, and approximately 20 ng DNA templates and ddH_2_O. Thermal cycling was performed with an initial denaturation at 94 °C for 3 min, followed by 24 cycles of denaturation at 94 °C for 5 s, annealing at 57 °C for 90 s, and extension at 72 °C for 10 s, with a final extension at 72 °C for 5 min. The concentration of the purified PCR products was quantified using a Qubit 3.0 Fluorometer (Thermo Fisher Scientific (Invitrogen), Waltham, MA, USA). Libraries were quantified and normalized to a concentration of 10 nM. Subsequently, the indexed libraries were pooled and loaded onto an Illumina MiSeq platform following the manufacturer’s instructions (Illumina, San Diego, CA, USA). The raw sequencing reads generated from the environmental sediments, body epidermis, foregut and hindgut of sea cucumber *H. leucospilota* were deposited in the NCBI Sequence Read Archive (SRA) under BioProject accession PRJNA1451367.

### 2.3. Sequence and Bacterial Community Analysis

Raw paired-end reads (2 × 300 bp, Illumina MiSeq, Illumina, Inc., San Diego, CA, USA) were demultiplexed using sample-specific barcodes, allowing up to one mismatch. Paired-end reads were merged using the fastq_mergepairs command in VSEARCH v1.9.6 [22] with default parameters. Merged reads were filtered with VSEARCH v1.9.6 to remove sequences containing ambiguous bases or with expected error rates > 1.0. Quality-filtered merged reads were dereplicated using VSEARCH (derep_fulllength), retaining sequence abundance information. High-quality sequences were processed to infer amplicon sequence variants (ASVs) using the UNOISE3 denoising algorithm (implemented in VSEARCH v1.9.6) via the cluster_unoise command, which corrects sequencing errors by mapping low-abundance variants to higher-abundance biological sequences based on abundance-rank and single-nucleotide distance criteria. Chimeric sequences were identified and removed using the UCHIME algorithm [23] as implemented in VSEARCH v1.9.6 [22], combining reference-based detection against the SILVA v138 database [24] with de novo chimera filtering. ASVs with a total abundance of fewer than 2 reads across all samples or detected in only a single sample were excluded from downstream analyses to minimize the influence of residual sequencing errors and potential contaminants.

Taxonomic assignment of ASV representative sequences was performed using a naive Bayesian classifier [25] trained on the SILVA v138 16S rRNA reference database [24] with a confidence threshold of 0.7. Relative abundances of taxa were summarized at phylum to genus levels for each sample. For phylogenetic diversity analyses, ASV representative sequences were aligned to the SILVA v138 SEED alignment using MAFFT (version 7.511) [26], and a maximum-likelihood phylogeny was inferred with FastTree (version 2.1.11) [27].

To standardize sequencing depth across samples for alpha- and beta-diversity comparisons, all samples were rarefied to 15,418 reads per sample (random seed = 42), the minimum sequencing depth across all samples. The resulting feature table was exported as a BIOM-format file [28].

### 2.4. Prediction of Bacterial Gene Function

Functional potential of the bacterial community was predicted from the ASV table using PICRUSt2 (version 2.3.0) [29]. The nearest-sequenced taxon index (NSTI) was calculated to assess the accuracy of the predictions. The weighted NSTI values were within the expected range for marine environmental samples, and no ASVs exceeded the default NSTI cutoff of 2.0, confirming acceptable prediction quality. ASV sequences were placed into a reference phylogeny, and nearest-sequenced taxon genome copies were used to infer KEGG Ortholog (KO) abundances. Pathway abundances were summarized at the KEGG module and MetaCyc levels to infer the overall metabolic functional profile of the bacterial community.

### 2.5. Statistical Analysis

The BIOM-format feature table described above was converted to a tab-delimited text format for downstream analysis in R (v3.6.1). Alpha diversity metrics, including Shannon and Chao1 indices, were calculated from rarefied ASV counts as described above. For beta diversity, unweighted UniFrac distances were computed from the rarefied ASV table and the phylogenetic tree described above, and visualized by PCoA. Additional ordination analyses were also performed, including PCA based on Hellinger-transformed relative abundances and Bray–Curtis PCoA and NMDS both based on Bray–Curtis dissimilarity of the rarefied ASV table. Analysis of similarities (ANOSIM) was performed on the Bray–Curtis dissimilarity matrix using 999 permutations to test for significant differences in bacterial community composition among compartments. Benjamini–Hochberg false discovery rate (FDR) correction was applied to adjust the *p*-values for the six pairwise comparisons. All visualizations and statistical validations were conducted in R using the vegan (version 2.5-6) [30] and ape packages (version 5.0) [31].

## 3. Results

### 3.1. Diversity of Bacterial Communities in Holothuria leucospilota

A total of 749,222 effective sequences were obtained from environmental sediments, epidermis, foregut, and hindgut of *H. leucospilota* from the South China Sea. The average numbers of effective sequences for sediments, epidermis, foregut, and hindgut were 49,956 ± 7914, 50,338 ± 10,990, 43,977 ± 3767, and 43,036 ± 5410, respectively. The effective sequences in environmental sediments (E1–E4) and epidermis (B1–B4) were more abundant than those in the foregut (F1–F4) and hindgut (H1–H4). The average number of ASVs for each compartment was 497 ± 14 (sample E), 242 ± 87 (sample B), 244 ± 42 (sample F), and 331 ± 24 (sample H) (Table 1). The number of ASVs in sediment samples was higher than in other samples. The proportion of sequences annotated against the database was over 99%, indicating that the proportion of undetected sequences in this study was negligible, and the results can reliably reflect the diversity of the bacterial community. The results of α-diversity indices showed that the richness of bacterial communities in environmental sediment samples was the highest, while the foregut samples exhibited the lowest Shannon diversity (3.96), followed by the epidermis (4.43). The hindgut showed higher diversity (5.38) than both the foregut and epidermis (Table 1).

**Figure 2:**
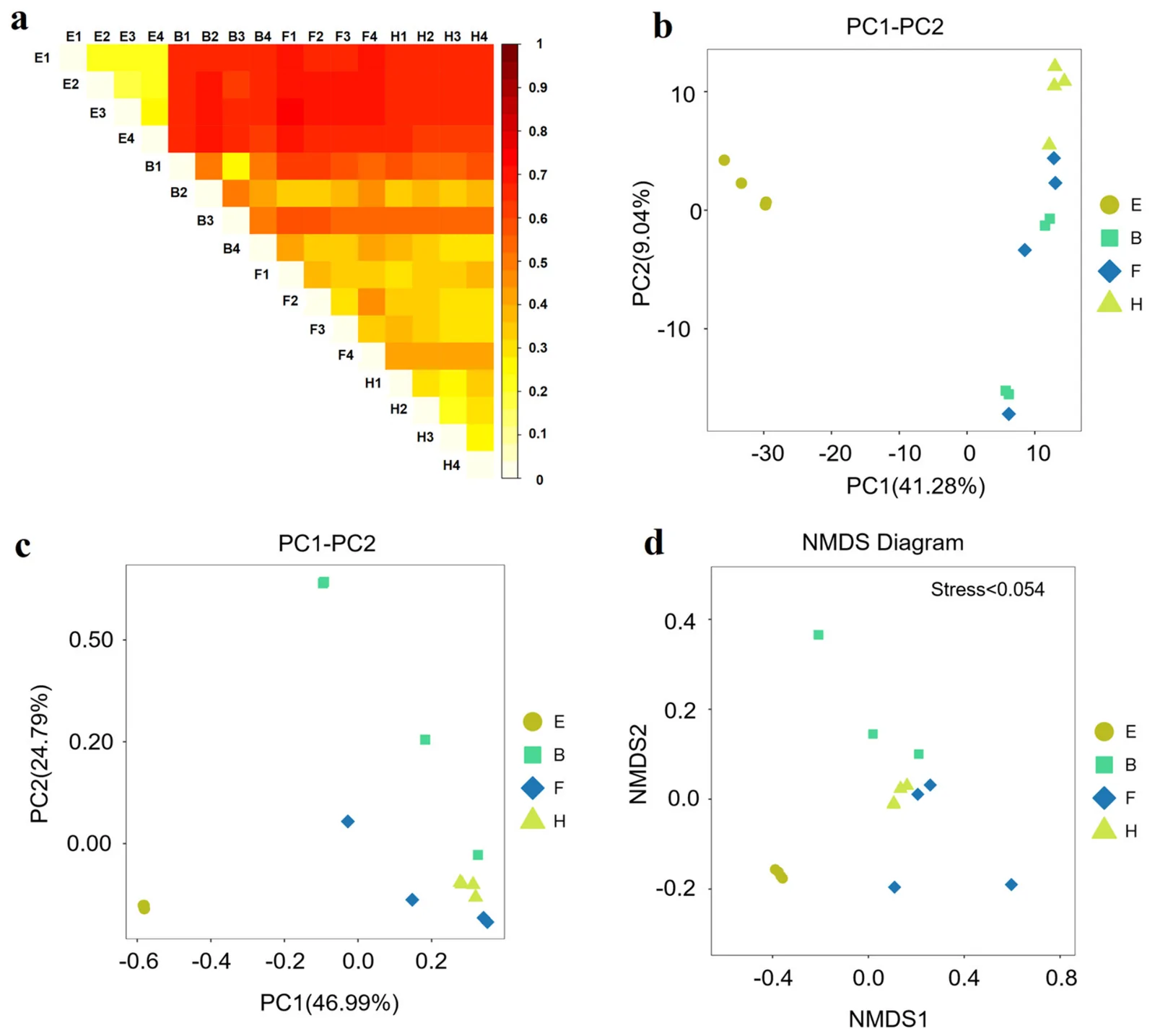
Unweighted unifrac analysis (**a**), principal component analysis (**b**), Principal co-ordinates analysis (**c**), and NMDS analysis (**d**) of bacteria communities in the different samples. E represents the environmental sediments. B, F and H represent body epidermis, foregut and hindgut of sea cucumber *H. leucospilota*, respectively.

Beta-diversity analysis was performed to assess differences in bacterial community composition among the four host compartments of *H. leucospilota*. The unweighted UniFrac analysis revealed a clear separation of bacterial communities along the sediment–epidermis–gut continuum (Figure 2a). Sediment samples formed a distinct cluster, indicating that the majority of environmental taxa were filtered out before colonizing host tissues. Notably, epidermal bacterial communities occupied an intermediate phylogenetic position between sediments and the gut, suggesting that the body surface acts as a primary selective barrier in associating bacterial transition from the environment to the host interior. Consistent with the unweighted UniFrac heatmap, PCoA and NMDS ordinations clearly separated sediment samples from host-associated compartments (Figure 2b–d). This pronounced structural divergence was underpinned by a distinct taxonomic partitioning of ASVs across compartments. Only 177 ASVs were shared among all four niches, whereas environmental sediments harbored the vast majority of unique ASVs (328). In stark contrast, the epidermis, foregut, and hindgut exhibited remarkably few unique ASVs (38, 23, and 28, respectively). This disparity suggests that host specificity is potentially driven by stringent filtering of the environmental bacterial community reservoir.

**Figure 3:**
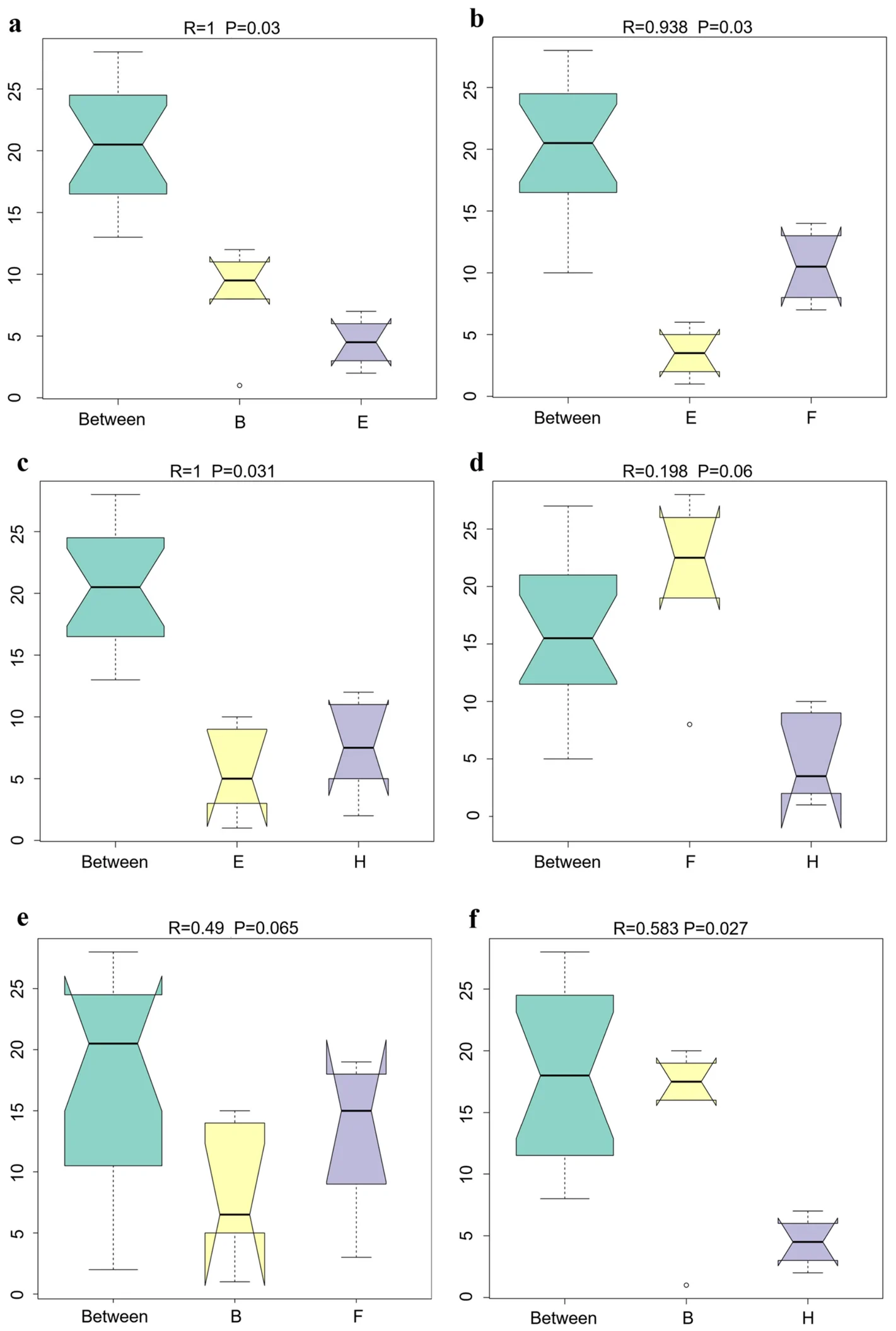
ANOSIM analysis of bacterial community dissimilarity among compartments. (**a**) Sediment (E) vs. Epidermis (B); (**b**) Sediment (E) vs. Foregut (F); (**c**) Sediment (E) vs. Hindgut (H); (**d**) Epidermis (B) vs. Foregut (F); (**e**) Epidermis (B) vs. Hindgut (H); (**f**) Foregut (F) vs. Hindgut (H). Each subplot displays the distribution of R-values (y-axis) based on 999 permutations; R-values close to 1 indicate complete separation between groups, whereas values near 0 indicate high similarity.

The total bacterial ASVs detected were classified into 28 phyla and 324 genera based on taxonomic assignment against the SILVA 138 database. ANOSIM was used to test for significant differences in bacterial community composition between compartments (Figure 3). Significant differences were observed between E and each host compartment (E vs. B: R = 1, *p* = 0.03; E vs. F: R = 0.938, *p* = 0.03; E vs. H: R = 1, *p* = 0.03) (Figure 3), revealing a distinct distribution of bacterial communities between sediments and host tissues. ANOSIM analysis showed no significant separation between foregut and hindgut communities (R = 0.198, *p* = 0.06), suggesting that their bacterial compositions were similar; however, we acknowledge that the absence of statistical significance does not prove community identity.

### 3.2. Bacterial Taxonomic Classification and Community Composition

**Figure 4:**
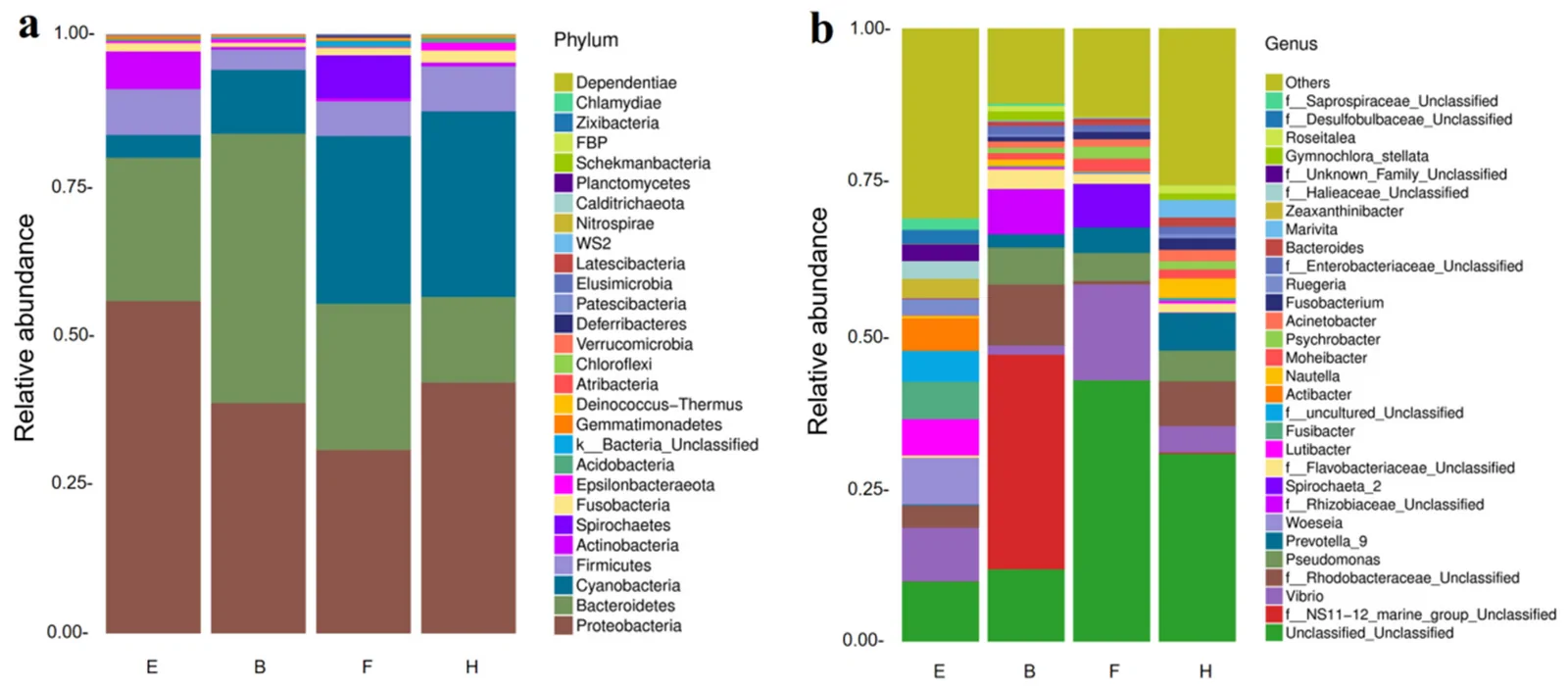
Relative abundance of bacteria at the phylum level (**a**). Relative abundance of bacteria at the genera level (**b**). E represents the environmental sediments. B, F and H represent body epidermis, foregut and hindgut of sea cucumber *H. leucospilota*, respectively. Each bar represents the mean relative abundance across the four biological replicates within each compartment.

At the phylum level, Proteobacteria, Bacteroidetes and Firmicutes were found to be the core microorganisms in all samples (Figure 4a), with relative abundances ranging from 55.47% (E) to 30.62% (F), from 44.95% (B) to 14.30% (H) and from 7.66% (E) to 3.41% (B), respectively, indicating that most of the bacterial species belonged to these taxa. Cyanobacteria accounted for a relatively large proportion in the intestine. Cyanobacteria accounted for a relatively large proportion, ranging from 30.96% (H) to 10.63% (B), but its proportion in E was lower (3.79%). In contrast, Actinobacteriota occurred more commonly in E (6.25%) than in other compartments (0.32–0.61%). Several phyla showed clear compositional differences between epidermal and gut samples: Fusobacteria [2.03% (H) to 0.73% (B)], Epsilonbacteraeota [1.31% (H) to 0.13% (F)] and Acidobacteria [0.14% (B) to 0.56% (H)], were more abundant in the gut than in the epidermis.

All bacterial ASVs recovered from the *H. leucospilota* compartments were taxonomically assigned to 256 known genera, with the remaining ASVs remaining unclassified at the genus level. The relative abundances of the 30 bacterial genera are shown in Figure 4b. The bacterial taxonomic composition differed across compartments of *H. leucospilota*. The dominant genus in epidermal samples was NS11-12 Marine Group Unclassified (35.01%), which was barely distributed in the other three compartments. *Vibrio* was the dominant genus in environmental sediments and foregut samples, accounting for 8.75% and 15.61%, respectively. The dominant genus in the hindgut samples was Rhodobacteraceae Unclassified (7.28%), followed by *Prevotella* (6.14%), *Pseudomonas* (5.04%), and *Vibrio* (4.29%). *Woeseia* (7.59%) was detected exclusively in environmental sediments, exemplifying environmental filtering, while *Spirochaeta* (7.12%) was almost entirely found in the foregut.

### 3.3. Predicted Functional Profiles of Bacterial Communities

**Figure 5:**
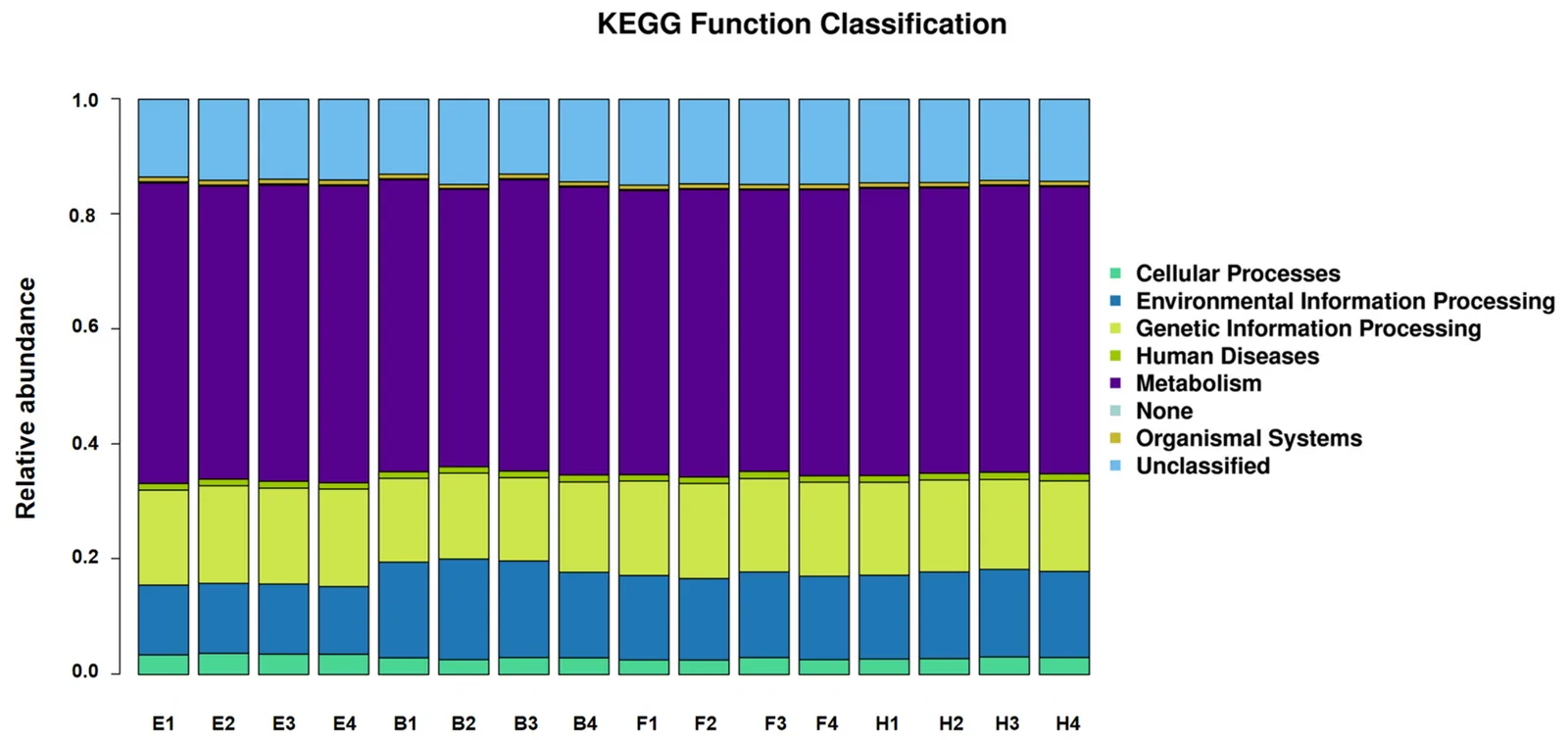
KEGG functional abundance in each sample. E represents the environmental sediments. B, F and H represent body epidermis, foregut and hindgut of sea cucumber *H. leucospilota*, respectively.

PICRUSt-based functional prediction was performed to infer the metabolic potential of bacterial communities. KEGG functional annotation revealed variations in the relative abundance of predicted functional categories across samples (Figure 5). A total of 8 functional categories and 41 KEGG level-2 functional categories were annotated. Among them, metabolism, genetic information processing, and environmental information processing accounted for the highest proportions. In the samples of body epidermis, the abundance of genes annotated to environmental information processing pathways was approximately 0.04 higher than in samples from other sites, which may be due to the continuous exposure of the body surface to the external environment. As the first line of defense, the epidermis may prioritize environmental adaptation over genetic information processing.

## 4. Discussion

A total of 324 bacterial genera were identified in this study, indicating that *H. leucospilota* possesses an unexpectedly diverse bacterial community. Alpha diversity analysis showed that the bacterial richness in environmental sediments was higher than that in the host compartments, with Shannon indices of 7.33 ± 0.11 (E), 4.43 ± 1.03 (B), 3.96 ± 0.4 (F), and 5.38 ± 0.51 (H), respectively. These results revealed a sharp ecological boundary between the environmental sediments and the host interior. Beta-diversity analysis further confirmed a sharp segregation of bacterial communities across compartments. The maximal ANOSIM R values (R = 1.0) indicated near-total discrimination between sediment bacterial communities and those of the epidermis, foregut, and hindgut, suggesting that environmental filtering rather than passive inoculation may shape bacterial communities assembly in *H. leucospilota*. We acknowledge that the small sample size (n = 4 per group) limits the statistical power of ANOSIM and that the observed patterns should be interpreted with caution. Consistent with this finding, a recent study on *Apostichopus japonicus* found that bacterial richness in the surrounding sediments was higher than those in the intestinal contents [20]. Similarly, from a study on tropical wild sea cucumbers *H. leucospilota*, it was found that bacteria abundance and diversity of foregut and hindgut in *H. leucospilota* were lower than those in the environmental sediments [32], which is consistent with our findings. Collectively, these results suggest that environmental filtering may be a key determinant of gut community assembly shaping compartment-specific bacterial communities in *H. leucospilota*. By comparing the bacteria across sediments, epidermis, foregut, and hindgut, we indicate a structured succession of bacterial assemblages influenced by increasingly stringent selective barriers.

At the phylum level, Proteobacteria, Bacteroidetes and Firmicutes were the core dominant phyla in all samples. Consistent with previous reports demonstrating the high abundance of Proteobacteria within sea cucumber gut compartments and associated sediments [20], our findings corroborate its prevalence from sediments to the gut. This broad distribution, likely driven by variations in sediment organic content [33,34,35], suggests localized environmental filtering. At the genus level, this filtering effect became more pronounced. *Woeseia* was unique to environmental sediments and is ubiquitously distributed in marine sediments [36]. *Vibrio* was the predominant genus in foregut samples. *Spirochaeta_2* was almost exclusively detected in the foregut. *Pseudomonas* and *Vibrio* were the dominant genera in the hindgut. Epidermal samples were dominated by the NS11-12 marine group (unclassified), which was minimally distributed in the other three compartments. The differential sorting of key taxa, exemplified by the exclusion of *Woeseia* and the selective retention of *Pseudomonas*, suggests that the observed variation in dominant genera likely reflects non-random, environmental filtering. In our study, the dominant bacterial genera in the intestines of *H. leucospilota* were *Vibrio*, unclassified* Rhodobacteraceae*, *Prevotella*, and *Pseudomonas*. While a previous culture-dependent study on *H. leucospilota* from Japanese coastal waters identified *Bacillus* and *Vibrio* as the dominant genera [37], these disparities likely stem from geographic variation in environmental bacterial communities. This aligns with our hypothesis that environmental filtering is a potential determinant of gut community assembly, wherein the distinct bacterial composition of local sediments shapes the selective colonization of the host gut.

A stepwise trajectory of compartment-specific differentiation can be inferred, wherein environmental exposure serves as the starting point, followed by selective screening at the body surface, and ultimately leading to microbial establishment within the intestine. This process may be shaped by the innate immune system comprising coelomocytes and humoral factors (such as lectins and antimicrobial peptides) [38,39]. One possible explanation for the strict confinement of *Woeseia* to sediments is that it may be sensitive to host-derived antimicrobial compounds or unable to adhere to the epidermal mucus layer [40]. However, this hypothesis requires direct experimental testing, as we did not measure host immune factors or bacterial adhesion properties in this study. *Vibrio* was highly abundant in sediments (8.75%) but less abundant on the body surface (1.49%), suggesting that epidermal immunity and chemical factors inhibit its attachment. However, its dominance in the foregut suggests that *Vibrio* may be introduced through sediment ingestion during feeding and may subsequently proliferate using host-derived nutrients. *Pseudomonas* was not detected in sediments but was consistently present on the body surface (6.04%) and in the intestines (foregut: 4.58%; hindgut: 5.04%), suggesting its role as a host-associated symbiont. Members of the genus *Pseudomonas* are known to exhibit diverse metabolic capabilities and have been reported to colonize marine host surfaces by exploiting local chemical microenvironments [41], suggesting that they may have established chemical ecological interactions for their hosts. Both *Pseudomonas* and *Vibrio* have been reported in other studies to exhibit antagonistic activity against aquatic pathogens [42,43]. It is therefore tempting to speculate that their coexistence in the intestinal tract might contribute to a disease-suppressive microenvironment. However, we did not assess antagonistic interactions or strain-level functions in our samples, and this hypothesis awaits experimental verification.

KEGG pathway analysis revealed that a total of eight functions were annotated across all samples, with the predominant functions being metabolism, genetic information processing, and environmental information processing. The high abundance of metabolic functions indicates that the gut bacterial communities are predicted to participate in the degradation and synthesis of various substrates such as carbohydrates, proteins, and lipids [44,45,46], potentially providing the host with critical metabolites including short-chain fatty acids and vitamins [47,48], while also contributing to the maintenance of intestinal homeostasis. Consistent with environmental filtering, epidermal bacterial communities were dominated by pathways related to environmental information processing. This functional specialization likely reflects the need for surface-associated bacteria to sense and respond to fluctuating external conditions, whereas gut bacterial communities showed a relative predominance of core metabolic pathways, reflecting selective pressures to exploit host-derived nutrients. This difference may stem from their role as the first line of defense between the host and the external environment, requiring continuous adaptation to environmental changes. Studies have indicated that environmental information processing, particularly signal transduction, is disrupted in sea cucumbers during heat stress [49], leading to bacterial community dysbiosis. This highlights the critical role of environmental signal perception in maintaining gut homeostasis, a principle consistent with our observation that compartment-specific environmental filtering may be a key driver of bacterial community structure. It should be noted that all functional profiles were predicted in silico from 16S rRNA data (KEGG), which reflects genetic potential rather than actual metabolic activity or direct inter-species cooperation; verification via shotgun metagenomics or in vitro co-culture is needed in future studies.

In this study, we acknowledge that all samples were collected from a single geographic location (Sanya coastal waters) during a single sampling month (August 2024). Consequently, the observed patterns of bacterial community differentiation and environmental filtering primarily reflect the local conditions at this specific site and time point. Seasonal variation, interannual fluctuations, and geographic heterogeneity in sediment composition, water temperature, and host population genetics may influence the bacterial assemblages of *H. leucospilota* across its distribution range. Therefore, extrapolation of our findings to the entire South China Sea or to the species as a whole should be made with caution. Broader spatiotemporal sampling across multiple seasons and locations will be necessary to assess the generality of the environmental filtering patterns reported here.

## 5. Conclusions

In conclusion, this study provides comprehensive evidence that the bacterial communities of *H. leucospilota* are spatially structured across environmental sediments, epidermis, foregut, and hindgut. We acknowledge that this study employed a cross-sectional design with each compartment sampled from different individuals, which precludes direct inference of a temporal colonization sequence. Moreover, the absence of community assembly null-model analyses means that stochastic processes cannot be formally excluded. Rather than passive inoculation, our findings are consistent with a stepwise environmental filtering process that may shape bacterial community assembly. The observed distinct taxonomic and functional profiles across compartments indicate that the epidermis serves as a critical interface for environmental sensing, whereas the gut bacterial communities are dominated by metabolic functions, particularly carbohydrate and amino acid metabolism. Although sediments represent a potential source pool for host-associated microbes, the strong filtering observed at the host interface suggests that sediment bacterial diversity alone does not determine gut community structure. Rather, our results highlight a functional partitioning strategy in *H. leucospilota*, where the separation of environmental sensing (epidermis) from nutrient acquisition (gut) may optimize the host’s adaptation to nutrient-poor benthic environments. Our study provides a descriptive baseline for understanding multi-compartment bacterial dynamics in *H. leucospilota*. These findings may serve as a reference for future hypothesis-driven research, including experimental assessments of host–microbe interactions and their relevance to aquaculture health management.

## Figures and Tables

**Table 1:** Comparison of bacterial sequences, the estimated ASVs richness, Chao1 index, and Shannon index of the bacterial 16S V4 sequences from the sequencing analysis. E represents the environmental sediments. B, F and H represent body epidermis, foregut and hindgut of sea cucumber *H. leucospilota*, respectively.

Samples	ASV	Chao 1	Shannon	Coverage
E1	486	533.63	7.43	0.996
E2	499	561.66	7.18	0.995
E3	486	540.24	7.32	0.996
E4	515	592.03	7.39	0.996
Average of E	497 ± 14	556.89 ± 26.30	7.33 ± 0.11	0.996 ± 0.001
B1	184	214.27	3.57	0.998
B2	226	230.11	4.82	0.999
B3	189	205.03	3.61	0.998
B4	369	403.07	5.70	0.997
Average of B	242 ± 87	263.12 ± 93.87	4.43 ± 1.03	0.998 ± 0.001
F1	224	239.62	4.49	0.998
F2	296	320.60	3.81	0.998
F3	257	289.80	3.55	0.998
F4	200	253.64	3.97	0.996
Average of F	244 ± 42	275.92 ± 36.53	3.96 ± 0.40	0.998 ± 0.001
H1	295	311.03	4.64	0.998
H2	344	362.50	5.48	0.998
H3	347	382.04	5.66	0.997
H4	337	353.73	5.74	0.998
Average of H	331 ± 24	352.33 ± 29.97	5.38 ± 0.51	0.998 ± 0.001

## Data Availability

The raw 16S rRNA gene sequencing reads generated in this study have been deposited in the NCBI Sequence Read Archive (SRA) under BioProject accession PRJNA1451367 and are freely available at https://www.ncbi.nlm.nih.gov/sra (accessed on 9 April 2026). The original contributions presented in this study are included in the article. Further inquiries can be directed to the corresponding authors.
